# To React or Not to React: The Dilemma of Fish Immune Systems Facing Myxozoan Infections

**DOI:** 10.3389/fimmu.2021.734238

**Published:** 2021-09-16

**Authors:** Astrid S. Holzer, M. Carla Piazzon, Damien Barrett, Jerri L. Bartholomew, Ariadna Sitjà-Bobadilla

**Affiliations:** ^1^Institute of Parasitology, Biology Centre of the Czech Academy of Sciences, České Budějovice, Czechia; ^2^Fish Pathology Group, Institute of Aquaculture Torre de la Sal - Consejo Superior de Investigaciones Científicas (IATS-CSIC), Castellón, Spain; ^3^Department of Microbiology, Oregon State University, Corvallis, OR, United States

**Keywords:** adaptive immunity, B lymphocytes, T lymphocytes, immunoglobulin, immune evasion, teleost, parasite, RNAseq

## Abstract

Myxozoans are microscopic, metazoan, obligate parasites, belonging to the phylum Cnidaria. In contrast to the free-living lifestyle of most members of this taxon, myxozoans have complex life cycles alternating between vertebrate and invertebrate hosts. Vertebrate hosts are primarily fish, although they are also reported from amphibians, reptiles, trematodes, mollusks, birds and mammals. Invertebrate hosts include annelids and bryozoans. Most myxozoans are not overtly pathogenic to fish hosts, but some are responsible for severe economic losses in fisheries and aquaculture. In both scenarios, the interaction between the parasite and the host immune system is key to explain such different outcomes of this relationship. Innate immune responses contribute to the resistance of certain fish strains and species, and the absence or low levels of some innate and regulatory factors explain the high pathogenicity of some infections. In many cases, immune evasion explains the absence of a host response and allows the parasite to proliferate covertly during the first stages of the infection. In some infections, the lack of an appropriate regulatory response results in an excessive inflammatory response, causing immunopathological consequences that are worse than inflicted by the parasite itself. This review will update the available information about the immune responses against Myxozoa, with special focus on T and B lymphocyte and immunoglobulin responses, how these immune effectors are modulated by different biotic and abiotic factors, and on the mechanisms of immune evasion targeting specific immune effectors. The current and future design of control strategies for myxozoan diseases is based on understanding this myxozoan-fish interaction, and immune-based strategies such as improvement of innate and specific factors through diets and additives, host genetic selection, passive immunization and vaccination, are starting to be considered.

## 1 Introduction

**Figure 1:**
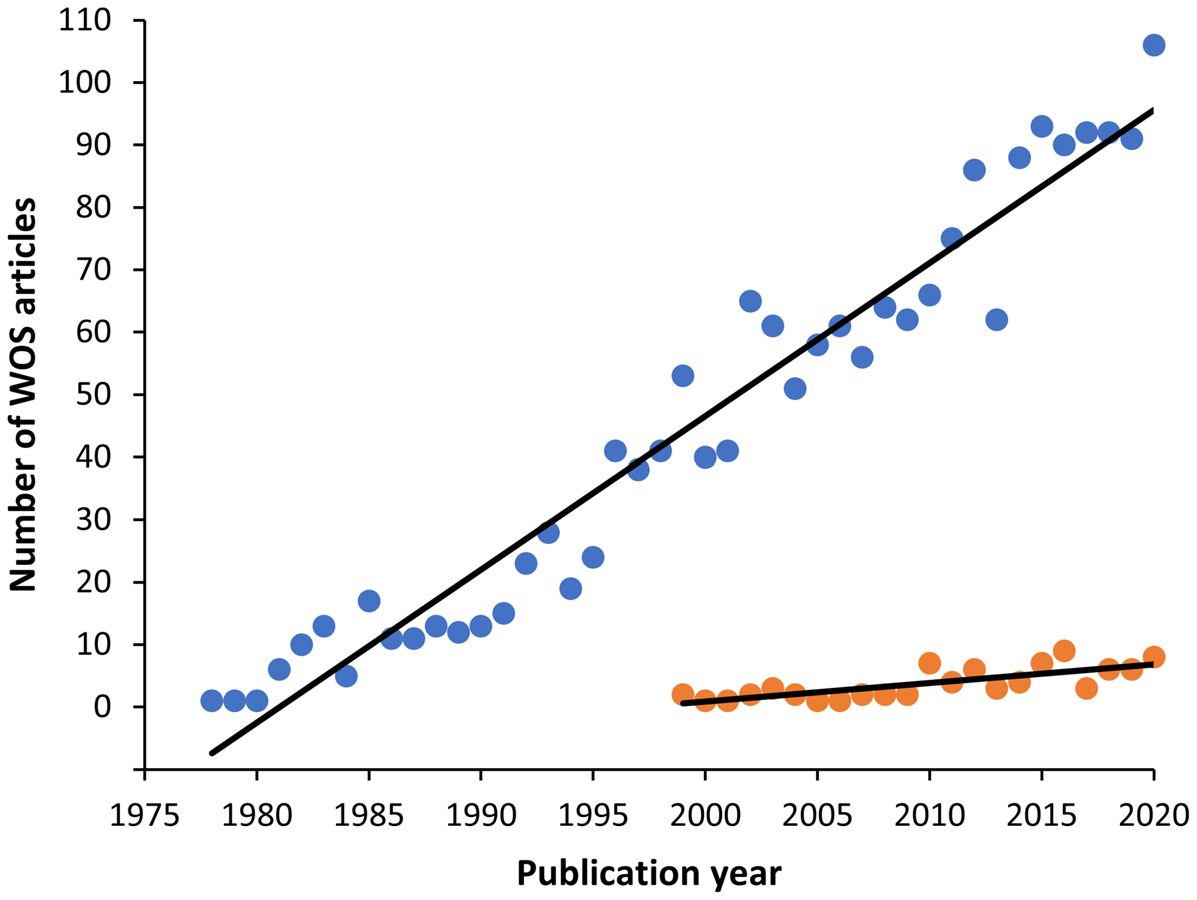
Graph showing the increasing trend of myxozoan publications found in the Web of Science (WOS) from 1900 to 2020. The search terms were: Myxozoa or Myxosporea or Actinosporea or Malacosporea (blue circles). The first article found in this database was in 1978. Among them, immunology related articles however appeared later in 1993, with a minor number, but also with an increasing trend (orange circles).

Myxozoans are obligate, microscopic, spore forming endoparasites, initially considered as Protozoa and long regarded as an enigmatic clade. Although cellular and molecular evidence clearly places the myxozoans as parasitic cnidarians, this obscure group is still not broadly recognized by biologists (1). Myxozoans demonstrate extreme morphological simplification and miniaturization as a result of their parasitic lifestyle (2). This subphylum comprises only two classes, Myxosporea and Malacosporea. Members of the former class use oligochaetes and polychaetes as definitive hosts and various vertebrates (mainly fish) and rarely invertebrates as secondary hosts. Members of Malacosporea use freshwater bryozoans and fish as hosts. Although studies on Myxozoans were first published in the late 1800s, a burst of publications has occurred in recent decades with the advent of “omic” technologies (**Figure 1**). This interest comes not only from the phylogenetic, evolutionary, and the still intriguing aspects of their basic biology, but also because some Myxozoans are responsible for severe economic losses in aquaculture and fisheries (3–8). Through millions of years of host-parasite evolution, Myxozoa have become masters in exploiting their hosts, some apparently causing little harm and others inflicting serious illness and mortalities, or endangering host reproduction (9). Infections can be coelozoic or histozoic and can occur in any tissue type of their fish hosts. Although generally considered extracellular parasites, some have intracellular stages that allow them to evade host defenses.

Among salmonids, the most devastating species are *Myxobolus cerebralis* infecting the cartilaginous tissue and brain, and causing whirling disease (10, 11), *Tetracapsuloides bryosalmonae*, the agent of proliferative kidney disease (PKD), producing a chronic immunopathological condition due to lymphocytic hyperplasia, hyperimmunoglobulinemia, and renal atrophy (12, 13), and *Ceratonova shasta* infecting the gastrointestinal tract and causing enteronecrosis (14). Examples among cyprinids are *Myxobolus wulli* (15) causing cystic liver disease, *Hoferellus carassi* causing kidney enlargement disease (KED) (16), *Myxobolus hunghuensis* causing pharyngeal myxobolosis (17), *Sphaerospora dykovae* producing swim bladder inflammation (SBI) (18), and *Sphaerospora molnari* that invades the skin or gill epithelia and multiplies in the blood prior to spore formation (19). In ictalurids, *Henneguya ictaluri* causes proliferative gill disease (PGD) (20). Among marine fish, the enteric parasites *Enteromyxum scophthalmi*, which results in sunken head syndrome and high mortalities in turbot, and *Enteromyxum leei*, which produces emaciative disease in more than 60 fish species (21) are important. *Myxobolus acanthogobii* produces skeletal deformities in *Seriola quinqueradiata* (22), and several *Kudoa* species cause muscular and brain/cardiac kudoasis (4, 23). In particular, *K. thyrsites* infects at least 35 marine fish species (24).

**Figure 2:**
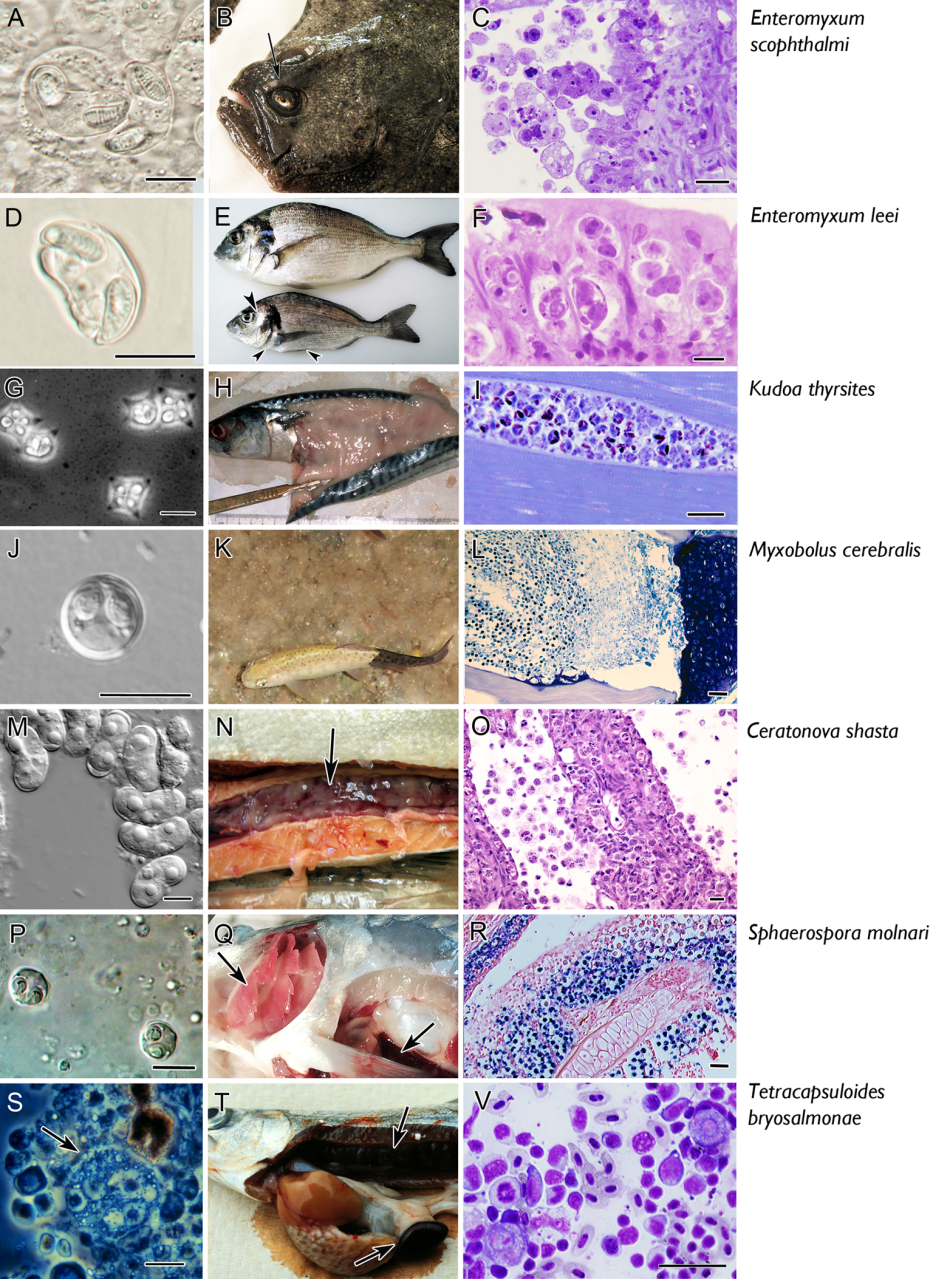
Micro and macro photographs of the main seven myxozoan species for which information is available on fish adaptive immune response. Pictures in the left column **(A, D, G, J, M, P, S)** fresh smears of myxospores, except for *T. bryosalmonae* in which a proliferative stage is shown **(S)**. Middle column refers to clinical signs: note the sunken head syndrome in turbot (arrow in **B**), the sunken belly and prominent head bones in gilthead sea bream (arrowheads in **E**), the myoliquefaction in a mackerel **(H)**, the black tail in rainbow trout **(K)**, the swollen and ascitic digestive tract in rainbow trout **(N)**, the pale gills and splenomegaly in common carp (arrows in **Q**), the swollen kidney and splenomegaly in rainbow trout (arrows in **T**). The right column depicts histopathological aspects. Catarrhal enteritis in turbot (**C**, Giemsa); invasion of the paracellular space of the gut (Giemsa, **F**); hypertrophy of myocytes (**I**, toluidine blue); invasion of the cartilage of rainbow trout (**L**, Giemsa); destruction of the intestine with detachment of stages to the lumen (**Q, H, E)**; sporogony in the gill epithelium [**R**, *in situ hybridisation* with parasites labelled in by VectorBlue, counterstained with Neutral red, according to Eszterbauer et al. (25)]; interstitial stages in a kidney imprint (**V**, Diff-Quick). Scale bars: 10 µm **(A, C, D, F, G, J, M, P, S)**; 20 µM **(I, O, V)**; 50 µM **(L, R)**. Illustrations courtesy of C. Zarza, ARC Skretting **(B)**; M. Kent, Oregon State University, USA **(G)**; S. Hallet, Oregon State University, USA **(K)**; S. Atkinson, Oregon State University, USA **(M)**; E. Eszterbauer, Hungarian Academy of Sciences **(P)**. The remaining figures are from the authors.

Control of myxozoan disease is problematic. There are currently no treatments or vaccines available in the market and only preventive and management strategies are in place. Among potential future strategies, exploitation of the fish immune system is one of the most promising. This has led to an increasing interest in deciphering aspects of the host-parasite relationship that relate to invasion, migration and replication of the parasite in the fish, and the host response at each of these stages of infection. However, the life cycles of only a few myxozoans are established in the laboratory, thus limiting the number of myxozoan-fish models for which relevant information about the immune response is available. This review summarizes what is known about adaptive immunity of the fish host in response to these few species, whose hosts and pathological effects are summarized in **Figure 2**.

Furthering our understanding of the fish immune response against myxozoans will require establishing their life cycles and their route of entry into the fish host. Since the milestone discovery of the first two-host life cycle for *M. cerebralis* (26), an alternation between vertebrate and invertebrate hosts has been described for 55 species. However, nearly all known life cycles involve freshwater fish, with only few described from marine species [see S1 in Holzer et al. (27)]. In the myxosporean cycle, the myxospore (the resistant and transmission stage to the annelid), is ingested by an annelid, and the released sporoplasm replicates in the gut epithelium, coelom or epidermis. After merogonial and gametogonial divisions, the parasite undergoes sporogenesis to produce actinospores. Actinospores (the transmission stage to the fish) released from the worm float in the water column and attach to the fish surface (skin or gills), and the released sporoplasms penetrate through the epithelium. Within the fish host, the pre-sporogonic stages migrate *via* the nervous or circulatory systems until they reach the final site of infection and develop into myxospores again (28–31). In contrast, malacosporeans develop either as vermiform or sac-like stages within the body cavity of bryozoans. For *T. bryosalmonae*, sacs produce malacospores which are released from the bryozoan. Upon encountering a fish, the sporoplasm penetrates *via* the skin and gills (32, 33). Within the fish host, *T. bryosalmonae* develops in the kidney interstitium and tubule lumina, where fish malacospores are formed and released to the water through the urine (34). One known exception to this two-host life cycle are species of *Enteromyxum*, which can be transmitted directly between fish hosts (21).

## 2 General Aspects of the Innate Immune Response Against Myxozoa

Studies on the immune response against parasites of humans and farmed animals are extensive, but despite this, vaccines for even the world’s major parasitic human diseases do not yet exist. Parasites represent many different taxonomic groups and their interaction with their hosts has many varied facets. To understand fish immune responses against myxozoan infections and the difficulties in developing vaccination-based control strategies, we start with a brief overview of what is known about the fish immune system.

Fish are one of the largest groups of vertebrates and the first animal group having both innate and adaptive immunity. Teleost and mammalian immune systems share a similar repertoire of molecules and cells involved in innate and adaptive immune responses [a full repertoire of T and B cell receptors, major histocompatibility complex (MHC) molecules, and immunoglobulins (Igs)] (35, 36), but there are several differences concerning both elements and functionality. For example, in the absence of bone marrow and lymph nodes in fish, the head kidney and the spleen are the main lymphohematopoietic organs. In addition, many aspects of adaptive immune function appear to have evolved independently in fish, with numerous teleost immune genes being at least duplicated (37, 38). Teleost fish have only three Ig isotypes: IgM, IgT/Z, and IgD (39), there is no switch of Igs in the secondary response, and the production of specific antibodies is temperature dependent (40, 41) (see section 3 for more details about the different functions of Igs). In the last decade our knowledge of the immune system of fish has notably increased (42–53), and in parallel, that of the immune responses against fish parasitic flagellates (54, 55), microsporidians (56), ciliates (57–60), helminths (61–64), amoeba (65, 66) and crustaceans (67–69). However, there are methodological difficulties that limit our understanding of fish immune responses in comparison to other vertebrates. As an example, the lack of fully validated monoclonal antibodies for cell surface receptors constrains differentiation of leucocyte subsets, especially for T cells. This technical constraint has hindered the isolation and characterization of Th1, Th2, Th17 and Treg cells.

To understand the fish immune responses against Myxozoa, we have to consider that they are multicellular organisms (as helminths) but are microscopic in size and may behave as protozoa in some aspects. In a generalized myxozoan infection [reviewed by Sitjà-Bobadilla et al. (70)], when the infective stages (actinospores, malacospores or proliferative stages of *Enteromyxum* sp.) first contact the fish host through mucosal surfaces (gills, skin, buccal cavity, or gastrointestinal tract), the innate immune system is automatically activated or they may pass unrecognized. Parasite exclusion can occur at the mucous barrier, which is comprised of a mesh of mucins in which commensal microbiota, lysozyme, lectins, complement, and Igs can interact. If the parasite survives this initial barrier, it encounters different cell types (macrophages, granulocytes including mast cells, B cells, T cells) and Igs at the subsequent mucosal layer. If the parasite successfully traverses the mucosal barrier, parasitic stages display different immune evasion strategies. Some proliferate and travel through the blood or the nerves to the final target tissue, others migrate to immunoprivileged sites such as eyes, gonads or brain. Some myxozoans may be eliminated during their blood passage by different cellular (granulocytes/mast cells) and humoral factors (lysozyme, complement, antiproteases, Igs), whereas others survive and even proliferate (70, 71). Once the parasite invades the target tissue, the host again engages cellular factors and immunoactivating and immunosuppressive cytokines. This interplay induces acute and chronic responses that, depending on the host-myxozoan model, may culminate in encapsulation and clearance of the parasite. However, some parasites may survive within the granuloma, or the granulomatous inflammation can lead to immunopathological consequences and even death of the host, with the subsequent release of the parasite to the environment [see (70) for more details].

Innate factors are also important for resistance of some fish species and fish strains against some myxozoan infections. The mechanisms involved in such complex phenomenon have been difficult to elucidate and studies comparing relative levels of resistance to infection and disease recognize that these mechanisms likely affect penetration and early establishment of the parasite, and they may have evolved differently among resistant species and strains of fish (72). Inter-specific differences in susceptibility to myxozoan diseases have been documented for *C. shasta* (72) and for *M. cerebralis* (73, 74) among salmonid species. Based on epizootiological data, Sugiyama et al. (75) indicated that amberjack (*Seriola dumerili*) was less susceptible to *Kudoa amamiensis* than yellowtail (*Seriola quinqueradiata*). Data obtained from PKD experimental infections indicate that the development of the parasite and the severity of the disease may vary with host salmonid species (76, 77). Interspecific differences have also been reported for *E. leei*, as some marine aquarium-reared (78) and several freshwater (79) fish are refractory to infection, and pathogenic effects differ substantially among susceptible species (21). Some immune factors have been suggested for these differences: in sharpsnout sea bream, the absence of lysozyme in either *E. leei*-infected or healthy animals (80, 81) may increase susceptibility to the high pathogenicity of this myxozoan in this host (80). Similarly, *E. scophthalmi* which is highly pathogenic for turbot, seems to be less harmful for sole (*Solea senegalensis*), as experimentally infected fish and cultured stocks have much lower infection levels than turbot, and infected fish did not show typical emaciated signs (82).

There are also clear examples of intra-specific differences in susceptibility to myxozoan diseases. Turbot stocks of different origin exhibited different susceptibility to *E. scophthalmi* in natural (83) and experimental (84) infections. Similarly, field and experimental data suggest that some gilthead sea bream stocks are partially resistant to *E. leei* (85, 86). There are also intra-specifics differences in susceptibility to *C. shasta* in several salmonids, a trait that has been exploited for disease management. Resistant strains of Chinook salmon (*Oncorhynchus tshawytscha*) are able to clear the parasite from the blood within two weeks, before infection is fully established in the intestine (31). Trends in cytokine expression and inflammatory cell recruitment suggest that susceptible fish react inappropriately to the infection, with resulting immunopathology that leads to host death. In rainbow trout (*Oncorhynchus mykiss*), inherited resistance to *C. shasta* is linked to multiple loci (87, 88), but protection can be overwhelmed by high and continuous parasite challenge (31). Rainbow trout strains resistant to *C. shasta* are susceptible to *M. cerebralis*, suggesting that different mechanisms might be specifically involved in the resistance to each myxozoan (89). In brown trout (*Salmo trutta*), variations in resistance to *T. bryosalmonae* and tolerance to the temperature-dependent disease it causes were detected among fish populations from two neighboring rivers with different water temperatures (90).

The innate host response to myxozoan infection, to react or not to react, is a double-edged sword. The absence of reaction can result in coexistence of the parasite with the host or in its death. Coexistence is the result of millions of years of host-parasite coevolution that lead to species-specific differences in tolerance/resistance patterns (77, 91). On the other hand, hyperreaction, as evidenced in some infections by an extremely high inflammatory profile, can be the true pathological player, as in PKD. The activation of innate factors is necessary to trigger the specific immune response, but in some occasions, it is dampened or delayed and thus does not stop the proliferation and invasion of the parasite, which can prove lethal for the host. In other cases, it is successful and infected fish recover and even acquire resistance to re-infection [see more details in section 6.2 and (92, 93)]. This acquired response provides possibilities for the control of myxozoan infections in aquaculture settings. Although information is only available for some fish species and many questions remain, we gather in the following sections the main advancements in our knowledge of acquired immunity evoked by myxozoan infections.

## 3 Specific Immune Responses in Fish

The adaptive immune response is mediated by both B and T lymphocytes (B and T cells). Fish T cells are comprised of CD8^+^ cytotoxic T lymphocytes (CTL) and CD4^+^ T helper (Th) cells (43, 94). Although functional research on the roles and differentiation states of T cells in fish is far less comprehensive than in mammals and exists only for a few model fish species, T cell surface antigens appear to be conserved across vertebrate taxa, and genomic analyses and gene expression studies suggest similar functions in fish (94–98). CTLs express the membrane bound glycoprotein CD8 and are able to kill infected host cells when activated by an antigen in a MHC class I context. Th cells express CD4 and secrete cytokines that regulate the action of other immune cells, including B cells. Once activated, fish CD4^+^ T cells appear to be capable of undergoing functional differentiation into the effector subtypes Th1, Th2, Th17, and Treg based upon the cytokine signals they receive (95, 97, 99). In fish and mammals, Th1 cells coordinate the immune response to intracellular pathogens by promoting macrophage activation and CTL proliferation. Their differentiation is driven by IL12 and IFNγ, along with the transcription factors T-bet, STAT1, and STAT4 (100–103). Th2 cells are associated with immunity to extracellular parasites, particularly helminths, and help promote B cell proliferation and antibody production (104, 105). IL4/13 (which Th2 cells also secrete) and transcription factors GATA3, STAT5, and STAT6 drive Th2 cell differentiation. In mammals, the Th1 and Th2 responses are known to cross-regulate each other, with IFNγ suppressing Th2 proliferation, and IL4 suppressing Th1 proliferation (106, 107). This mechanism appears to be conserved in fish as well (108). Less is known about the function of Th17 cells in fish, although they are believed to play a similar role to their mammalian counterparts in mucosal immunity to extracellular bacteria and fungi. Th17 cells secrete IL17, IL21, and IL22 and are regulated by transcription factors RORγT and STAT3 (97). Regulatory T cells (Treg), as their name implies, help regulate the immune response by producing the anti-inflammatory cytokines IL10 and TGFβ, and they express the master transcription factor FOXP3 (109).

### 3.1 T Cell Response to Myxozoan Infection

While myxozoans infect a wide range of fish species and inhabit a diverse set of tissues, a strong Th1 polarization in the fish host appears to be common. Our knowledge of the host immune response primarily stems from gene expression studies, with fewer functional studies conducted due to a lack of fish-specific antibodies and reagents. However, some clear trends have emerged, including the upregulation of *ifnγ* in response to myxozoan infection, often followed by a massive spike in *il10* expression. Resistance to disease is associated with proper T cell activation, while disease pathology is associated with a dysregulated, and often excessive T cell response. Below we summarize what is known about T cell responses and how they contribute to resistance or immunopathology in model myxozoan systems.

Salmon and trout infected with *C. shasta* have a pronounced upregulation of *ifnγ* in their intestine (110, 111). This strong inflammatory response leads to severe intestinal inflammation, with lymphocytes infiltrating the infected tissues (112). This appears to be a maladaptive host response that contributes to host tissue damage and parasite proliferation (113). An RNA-seq time series found that in both resistant and susceptible steelhead trout infected with *C. shasta*, there was an initial downregulation of immune genes, particularly those associated with the Ifnγ signaling pathway, possibly indicating parasite-induced immunosuppression. The lack of response to the parasite continued in susceptible fish until the parasite was proliferating in the intestine and causing pathology. At this time the susceptible fish showed a vigorous Th1 polarization with significantly increased expression of *ifnγ* and *il12*, along with the Th1 transcription factors *t-bet* and *stat1* (114). Susceptible fish also showed upregulation of Th2 response cytokines (*il4/13*) as well as the master transcriptional regulator of Th2 differentiation, *gata3.* This response was ineffective at best, and likely contributed to host pathology as parasite numbers continued to increase exponentially and the intestinal structure of these fish rapidly broke down. A marked decline in expression of Th17 cell markers (*il17*, *stat3*, *rorγt*) followed, however it is unclear if that is directly related to *C. shasta* infection or if it is a by-product of the intestinal barrier breaking down. In contrast, resistant fish responded rapidly with upregulation of genes for innate immune receptors, including *nlrc5*, and induction of Th1 markers in the intestine, suggesting that early specific recognition of *C. shasta* is a critical factor in resistance (114). No decline in Th17 markers was observed, and these fish were better able to maintain their intestinal structure in the face of parasite proliferation. A shift towards Th2 polarization may be occurring late in the infection in resistant fish, with elevated expression of *il4/13, gata3*, and *stat5*, in addition to Th1 markers *ifnγ*, *il12*, and *tbet.* Given that the Th2 cells are classically associated with the immune response to extracellular parasites, their role in the immune response to myxozoan infections warrants further study.

Similarly, rainbow trout infected with *M. cerebralis* showed increased expression of *ifnγ*, and its induction occurred more rapidly in fish that are resistant to the disease (115). Resistant fish also had consistently higher expression of *stat3* in their caudal fin tissue compared to susceptible fish, and it was proposed that Th17 cells may play a role in combating *M. cerebralis* at the epithelia. A later study utilizing flow cytometry found that resistant fish had a much stronger T cell response compared to susceptible fish, with increased CD8^+^ and CD8^-^ (likely CD4^+^) T cell populations in the caudal fin, head kidney, and spleen (91).

In *T. bryosalmonae*-infected rainbow trout, gene expression profiling has shown that *cd4* and *cd8* transcripts are positively correlated with parasite burden (77, 116). A general Th1-like polarization is observed in these infections, with upregulation of *ifnγ* and *tbet* (117). However, Th17 profiles were also observed coupled with upregulation of *il10* and *il6*. This mixed profile was proposed as a dysregulated T cell response induced by the parasite infection (116). Depending on the environmental conditions, a more complex Th response has been observed, which will be discussed in more detail in section 4.

Korytář et al. (118) examined the cellular and humoral immune response of *S. molnari*-infected carp over a 63-day period and observed a mild systemic response in the head kidney during the initial stages of the infection, with increased expression of pro-inflammatory cytokines, including *ifnγ*. A peak in parasite numbers at 28 days post-infection was followed by a massive increase in the number of lymphocytes detected in the blood and a spike in *il10* expression (up to 1456-fold) at 56 days post-infection. As the authors noted, this may represent a host-induced attempt to mitigate damaging inflammation, or a parasite-induced form of immune evasion.

The response of Gibel carp (*Carassius auratus gibelio*) to *M. hunghuensis* was investigated by RNA-seq analysis of mildly- and severely-infected pharynx tissue (119). Increased expression of chemokine and chemokine receptors, along with T cell marker *cd3d*, indicate that leukocytes are recruited to the infection site. A mixed Th response appears to be occurring, with upregulation of both *ifnγ* and *stat1* (Th1), as well as *il4/13a* and *gata3* (Th2), although functional studies will need to be conducted to determine which Th subsets are actually present.

A clear role for T cells in resistance to myxozoan disease comes from Atlantic salmon (*Salmo salar*) infected with *K. thyrsites*. Atlantic salmon are able to resolve the infection and acquire a protective immunity to reinfection (120). Utilizing immunohistochemistry and targeted (RT-qPCR) transcriptomic data, Braden et al. identified a population of CD8α^+^ cells responding at the infection site in resistant fish (121). This correlated with increased expression of *il12*, *cd8*, and NK-lysin, an effector peptide produced by cytotoxic T cells, indicating that some of these cells were cytotoxic T cells.

The specific host responses to *Enteromyxum* infections are well-studied and indicate a clear regulation of T cell populations at both the local and system levels. Gilthead sea bream (*Sparus aurata)* infected with *E. leei* have increased expression of *cd4* and *cd8* transcripts along with pan T cell markers *zap70* and *cd3ϛ* in their anterior intestine (site of infection) with a concomitant downregulation of those genes occurring in the head kidney, that may indicate T cell migration towards the infected tissue. Immunohistochemistry (using cross-reacting anti-Zap70 antibodies) along with BrdU immunolabelling indicated that local proliferation of T cells likely did not cause the increase in T cells observed at the infection site, supporting T cell migration. In the intestine, the increased expression of CTL, Th1, and Th2 markers indicate the local activation of T cell responses, which may be kept in balance by the simultaneous upregulation of regulatory cytokines (*il10* and *il6*). This reaction occurs more strongly in the anterior part of the intestines whereas in the posterior intestine a more dysregulation profile, including Th17 cells markers, is evident (122). The similarities between these dysregulated T cell responses in *T. bryosalmonae*-infected rainbow trout (116) and *E. leei* infected gilthead sea bream (122) are noteworthy.

In turbot enteromyxosis, differential T cell responses have been inferred from untargeted transcriptomic analyses. The early stages of the infection are characterized by upregulation of Th1 markers at the local and systemic level (123). However, the advanced stages of the infection are characterized by lymphocyte depletion and downregulation of genes related to T cell activity in the lymphohematopoietic tissues (124–126). Interestingly, a decline in thymic function with loss of cellularity was revealed by genomic and morphopathological studies of infected turbot and appeared to be a consequence of infection and malnutrition (127). In general, turbot enteromyxosis seems to inhibit or depress the host’s ability to mount a proper adaptive immune response, which explains the high susceptibility and pathology associated with the disease (128).

### 3.2 B Cell Response/Antibody Production

Among the three distinct Ig isotypes of fish, IgM is generally considered to be involved in system immunity, with IgM^+^ B cells being dominant in the blood and systemic lymphoid organs (head kidney and spleen) and their proliferation in these sites increases during infection (129, 130). The fish-specific isotype IgT (referred to as IgZ in carp or zebrafish) is relatively recently described and appears to be primarily involved in mucosal immunity (131), however it may also play an important role in systemic responses (129). The role of IgD remains unknown, although B cells exclusively expressing IgD have been found in channel catfish blood (132) and in rainbow trout gills, where they appeared to respond to viral infections (133). Myxozoan infections are diverse in their infection route, target tissue and disease presentation, so it is not surprising that host responses to infection differ. In some myxozoan infections, the specific antibody response is delayed as a result of parasite migration using protected routes (e.g. *M. cerebralis*) (see section 5). In chronic or prolonged infections, there is generally increasing expression of IgT and IgM transcripts and evidence that protective antibodies are produced that are effective in delaying disease progression, reducing disease severity, and protecting from future infection (e.g. *T. byrosalmonae*, *S. molnari*, *E. leei* in sea bream, nonlethal strains of *C. shasta* or *C. shasta* infections in resistant fish strains, *K. thrysites, M. hunghuensis*). However, when infections are acute (e.g. enteromyxosis in turbot, virulent strains of *C. shasta*), there is little evidence for a role for B cells. Below we describe the specific responses for our model myxozoans.

**Figure 3:**
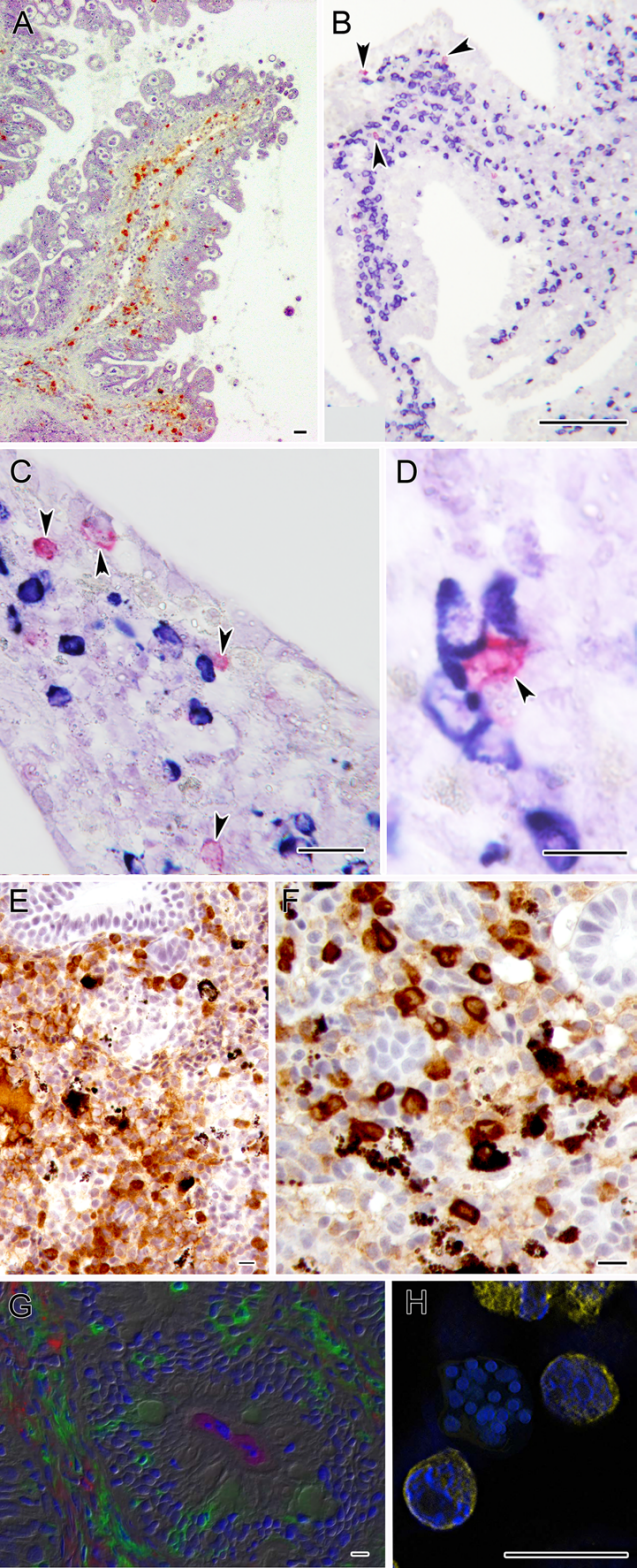
Microphotographs depicting the specific immune response against some myxozoans. **(A)** Turbot intestine infected by *E. scophthalmi* showing abundant IgM+ cells in the lamina propria submucosa, immunostained with a monoclonal antibody. **(B–D)** Panoramic and close ups of gilthead sea bream intestines infected with *E. leei* showing abundant IgM (blue) and IgT (magenta) positive cells. RNA-*in situ* hybridization (RNA-ISH) was used to detect transcripts of IgM and IgT as indicated in Picard-Sánchez et al. (134). **(E)** Rainbow trout kidney infected with *T. bryosalmonae* showing abundant IgM+ cells and **(F)** IgT+ positive cells. Sections were immunostained as indicated in Abos et al. (135). **(G)** Large accumulations of IgT+ B cells in the gut lamina propria and epithelium of rainbow trout surviving infection by *C. shasta*. Immunofluorescence staining of a gut cryosection from rainbow trout, three months post-infection with *C. shasta*. Cryosection was stained for IgM (red), IgT(green) and *C. shasta* (magenta); nuclei were stained with DAPI (blue). Parasites can be seen within the gut lumen. **(H)** *S. molnari* multicellular blood stage surrounded by B cells labelled for IgM (yellow) and stained with DAPI (blue). Scale bars: 10 µm **(D-H)**; 20 µm **(A, C)**; 100 µm **(B)**. Illustrations courtesy of R. Bermúdez, USC, Spain **(A)**; C. Tafalla, INIA, Spain **(E, F)**; O. Sunyer, University of Pennsylvania, USA **(G)**. The remaining figures are from the authors.

The role of IgT as a mucosal antibody was first described in *C. shasta*-infected rainbow trout (131). A significant increase in IgT and IgT^+^ B cells was observed in the intestines of infected fish (**Figure 3**), whereas IgM and IgM^+^ B cells were found in the blood. These antibodies were demonstrated to be *C. shasta*-specific using ELISA. It should be noted that although these fish were known to be susceptible to *C. shasta*, they were likely infected with a chronic, nonlethal parasite genotype that allowed time for the fish to respond to the infection. In infections of resistant and susceptible Chinook salmon with a more virulent parasite genotype, a marked increase in the number of Ig^+^ cells were found in the intestine of susceptible compared to resistant fish strains (110). Susceptible fish, which suffered 100% mortality, also had higher expression of *ifnγ*, *il6*, and *il10*, indicating an overactive and dysregulated adaptive immune response. This is similar to what was observed in acute infections of susceptible steelhead, where B cell markers *blimp1*, *cd22*, and Ig transcripts were significantly increased along with *ifnγ*, *il6*, and *il10* (114). In a subsequent study that examined acute infections in resistant and susceptible steelhead, the resistant fish had higher expression of *IgM* (14 versus 2-fold) and *IgT* (337 versus 65-fold) compared to susceptible fish, at 21 days post infection (dpi) (88).

Early studies of the adaptive response to *M. cerebralis* that attempted to demonstrate presence of specific antibodies relied on immunohistochemical methods to detect mature spores (136, 137). As these are the final stages of infection in the fish, it is perhaps not surprising that results of these reports are inconsistent. Detection of specific antibodies to actinospore or myxospore stage antigens was demonstrated in a later study, but the response was highly variable and without a repeated pattern of antigen recognition, and injection of serum collected from infected trout into naive fish provided only marginal protection against challenge (73). Infected fish were able to resist parasite invasion of the epidermis beginning as early as 35 days after initial exposure, suggesting that naturally produced antibodies may provide protection to a second exposure (73). However, protection from re-infection occurred only in fish that had developed infections with cartilage lesions. A delayed antibody response to *M. cerebralis* was also demonstrated by Ryce et al. (138), suggesting that the host is not able to respond to the infection until it causes sufficient damage to cartilage tissue to allow its exposure to host defenses. Whether this protection is greater than that afforded by the age-dependent process of ossification of the cartilage (and thus removal of the food source) is unclear.

PKD in rainbow trout is characterized by a dysregulated B cell response with Ig transcripts being strongly upregulated and highly correlated with kidney pathology (116). This upregulation was confirmed at the protein level, with all three Ig isotypes being upregulated in the kidney of infected rainbow trout (135). Interestingly, IgT was found to be the dominant isotype responding to the infection. IgT^+^ B cells were the main B cell subset proliferating in the kidney and the extrasporogonic stages of *T. bryosalmonae* were coated in IgT. As the authors noted, this may indicate that IgT plays a role in systemic immunity to parasites/myxozoans, similar to mammalian IgE.

In *S. molnari* infections of common carp (118), a significant increase in B cells and IgM levels is first observed 14-21 dpi, coinciding with the onset of the parasite’s proliferative blood stage phase. IgM (membrane bound form) expression increased steadily until 42 dpi when it reached more than 14-fold expression in the head kidney of individual fish. IgM^+^ B cell numbers in peripheral blood showed up to 60-fold increase, between 42 and 56 dpi. The authors demonstrated the acquisition of specific immunity of carp against *S. molnari*, but the low titer of specific antibodies in combination with an extremely high number of circulating B cells with high expression of *IgM* points to a possible hypergammaglobulinemia (118). It was shown that the acquisition of specific immunity to *S. molnari* in common carp is essential to control parasitemia levels in the blood as the suppression of B cell numbers resulted in massive parasite proliferation, hemolytic anemia and death due to parasites feeding on red blood cells (139).

In enteromyxosis of turbot, fish are sometimes able to survive the infection and acquire protective immunity to reinfection. Using immunohistochemistry and ELISA, this was shown to involve parasite-specific IgM (140). The number of IgM^+^ cells in the intestine increased over the course of the infection while decreasing in the spleen and kidney (124), indicating that these cells may be migrating to the infection site, similar to what is observed for T cells during gilthead sea bream enteromyxosis (122). However, most evidence indicates that the humoral response during turbot enteromyxosis is generally delayed and ineffective (84, 124, 141). RNA-seq analysis of severely infected turbot tissue found a similar pattern, where a global depression of genes involved in acquired immunity were downregulated in the spleen and head kidney, while immunoglobulin-related genes were upregulated in the pyloric caeca (126). A follow-up RNA-seq analysis of the same three organs during the early stages of enteromyxosis found little evidence for the activation of B cells, with the immune response at this stage primarily revolving around IFN-signaling pathways (123). The role of IgT in this host-parasite model has been poorly studied.

In gilthead sea bream, *E. leei* infections induced an increase in *IgM* and *IgT* transcripts, with a stronger effect observed in the posterior intestine, the main target site for this parasite (71, 130). Immunohistochemistry studies revealed an increased presence of lymphocyte-like cells in infected intestines, of which some were characterized by immunohistochemistry as IgM^+^ cells (71). On the other hand, the IgM^-^ population of lymphocytes was proposed to be, at least in part, IgT^+^ B cells. Regretfully, no IgT immunohistochemistry could be performed and this point is, to date, only supported by transcriptomic data (130). Morphological observations indicated that part of the IgM^+^ populations in the intestine were plasma cells (71), which was supported by the transcriptional increase of both soluble and membrane forms of *IgT* and *IgM* in infected animals (130). In addition, long-term infection with *E. leei* induced an increase in total serum IgM and IgT, however, whether these circulating antibodies were parasite-specific or not remains to be determined (130). IgD transcripts were not significantly regulated in this host-parasite model (122).

In addition to the more well studied systems, increased expression of *IgM* and *IgT* has been shown in Atlantic salmon infected with *K. thyrsites* (121) and Gibel carp infected with *M. hunghuensis* (119). This supports the evidence above that both IgM and IgT have conserved roles in the immune response to myxozoan infections. For many of the host-parasite systems studied, it is evident that fish are mounting an antibody response, but it is unclear if this is effective in controlling the infection or if it is simply contributing to host pathology.

## 4 Variation of B/T Cell Responses

Developing a clear picture of the adaptive immune response to myxozoan infection is complicated by the effects of temperature, differences in host susceptibility to disease, and variations in host specificity and virulence between parasite genotypes. In this section we review what is known about the effects of these parameters on the host immune response.

### 4.1 Temperature Effects

As poikilothermic organisms, the body temperature and physiology of fish are directly tied to ambient water temperatures that may change by more than 10°C due to seasonal variation, fish migration, or microclimates (142, 143). Immune response kinetics are directly influenced by temperature and thermal stress may inhibit or suppress the host immune system (144), altering the nature or course of the immune response (145). The adaptive immune response is more heavily influenced by temperature and fish appear to rely more on innate immunity at lower temperatures (146–148). In addition to the influence of temperature on the fish immune response, temperature influences the rate of parasite development in the fish and invertebrate host (149, 150), as well as transmission between hosts (150, 151). Although there are numerous studies that have examined the effects of temperature on myxozoan disease, only a few of these, described below, have examined the host response during these infections.

Even subtle temperature changes can have a profound impact on the host immune response to myxozoan infection. Bailey et al. exposed rainbow trout to *T. bryosalmonae* at 12°C and 15°C and measured immune gene expression over a 7-week period (152). The fish at 12°C had a significantly lower pathogen load with an increase in both lymphocytes and Th1 markers in the kidney. At 15°C, the pathogen burden was much higher, and a prominent B cell response was evident along with marked upregulation of Th2 markers and *il10*. The difference in immune response between temperature regimes remained even when fish with similar parasite burdens were compared. While temperature-dependent changes in parasite replication or virulence cannot be ruled out, this suggests that at higher temperatures the fish host employs a vigorous adaptive immune response that is maladaptive and contributes to the chronic immunopathology observed in rainbow trout PKD. In gilthead sea bream, high water temperatures increased the prevalence of *E. leei* infection, but at the same time induced a higher production of specific antibodies (IgM), thus limiting the progression of the infection along the intestine (153).

The differential host and parasite responses to temperature highlight the importance of considering this factor when researching or applying vaccination or treatment strategies. However, when considering the effects of temperature on myxozoan disease in a fish population is important to understand that temperature influences all aspects of the parasite life cycle.

### 4.2 Differences in Host Susceptibility to Disease

The phenotype of the fish host also influences the adaptive immune response to myxozoan infection. Differences in the timing and magnitude of the response are observed between fish with different innate resistance levels, as discussed earlier for *C. shasta* and *M. cerebralis* infections. The age of the host at the time of infection also leads to immune-related differences in response. When different age classes of rainbow trout (fish less than 1 year old, and fish between 1-2 years old) are exposed to *T. bryosalmonae*, younger fish experience much higher disease severity (154). This corresponds with significantly higher expression of *il10* and B cell master regulator *blimp1*, relative to older fish. While it cannot be determined if elevated expression of these genes is the cause, or product, of increased disease severity, overproduction of *il10* and B cells/immunoglobulins appears to be highly correlated with disease pathology in several fish-myxozoan systems.

### 4.3 Intra-Species Differences in Parasite Virulence

Intra-species differences in virulence are documented for at least one myxozoan species, with distinct host immune responses observed between infections by low and high virulence parasite strains. *C. shasta* exists as a species complex, with three distinct genotypes that vary in their host specificity and virulence (155, 156). *C. shasta* genotype 0 causes chronic, asymptomatic infections in rainbow trout and steelhead. Genotypes I (infects Chinook salmon), and II (infects Coho salmon, rainbow trout, and steelhead) are associated with acute, and often fatal, infections in their respective hosts. When susceptible Chinook salmon are infected with virulent genotype I, a systemic immune response is observed with increased gene expression of *ifnγ*, *il6*, and *il10* in the spleen (111). On the other hand, infection with the less virulent genotype II causes a localized immune response with increased expression of *ifnγ*, *il6*, and *il10* along with *IgM* and *IgT* in the intestine. A comparison of susceptible rainbow trout infected with either genotype 0 (chronic infection) or genotype II (acute infection) found significant differences in immunoglobulin production and cytokine expression in the intestine (113). Chronic infections are characterized by mild upregulation of *ifnγ* followed by an increase in *il10*. Acute infections resulted in significantly stronger induction of *ifnγ* along with the pro-inflammatory cytokines *il6* and *il8*. A massive spike in *il10* expression was also observed in acute infections, with an up to 200,000-fold increase 15 days after exposure. With regard to immunoglobulin production, chronic infections resulted in a moderate increase in IgM and IgT production at 29 days. In acute infections, *IgT* was upregulated by day 7 and continued to increase until 29 days (~700-fold). *IgM* expression followed a similar trend but was more moderately upregulated with a maximum ~52-fold increase observed.

## 5 Mechanisms of Immune Evasion Targeting Specific Immune Effectors

The longevity of these parasites in the face of the destructive potential of the immune system is remarkable and is most readily explained by the parasites’ ability to interfere with or evade host defenses. Parasites are undoubtedly notorious immunomodulators (157) and have evolved a wide array of effective immune evasion strategies (158, 159). The latter include e.g. avoidance of recognition during invasion by sequestering recognition tags (PAMPs), modification of antigens during parasite development, interference with antigen presentation or with the host’s immune response signaling network. The long-term persistence of some myxozoan infections in fish [e.g. *T. bryosalmonae* (154, 160), *S. molnari* (118), *C. shasta* genotype 0 (161)] despite the ability of fish to generate a specific antibody response, indicates that myxozoans, like more evolved metazoan parasites, effectively avoid or modulate immune responses. Although the underlying mechanisms are poorly understood, several reports allow for insights into the different strategies of myxozoans.

### 5.1 Myxozoans “Under Cover”: Immunoprivileged Sites and Intracellular Disguises

**Figure 4:**
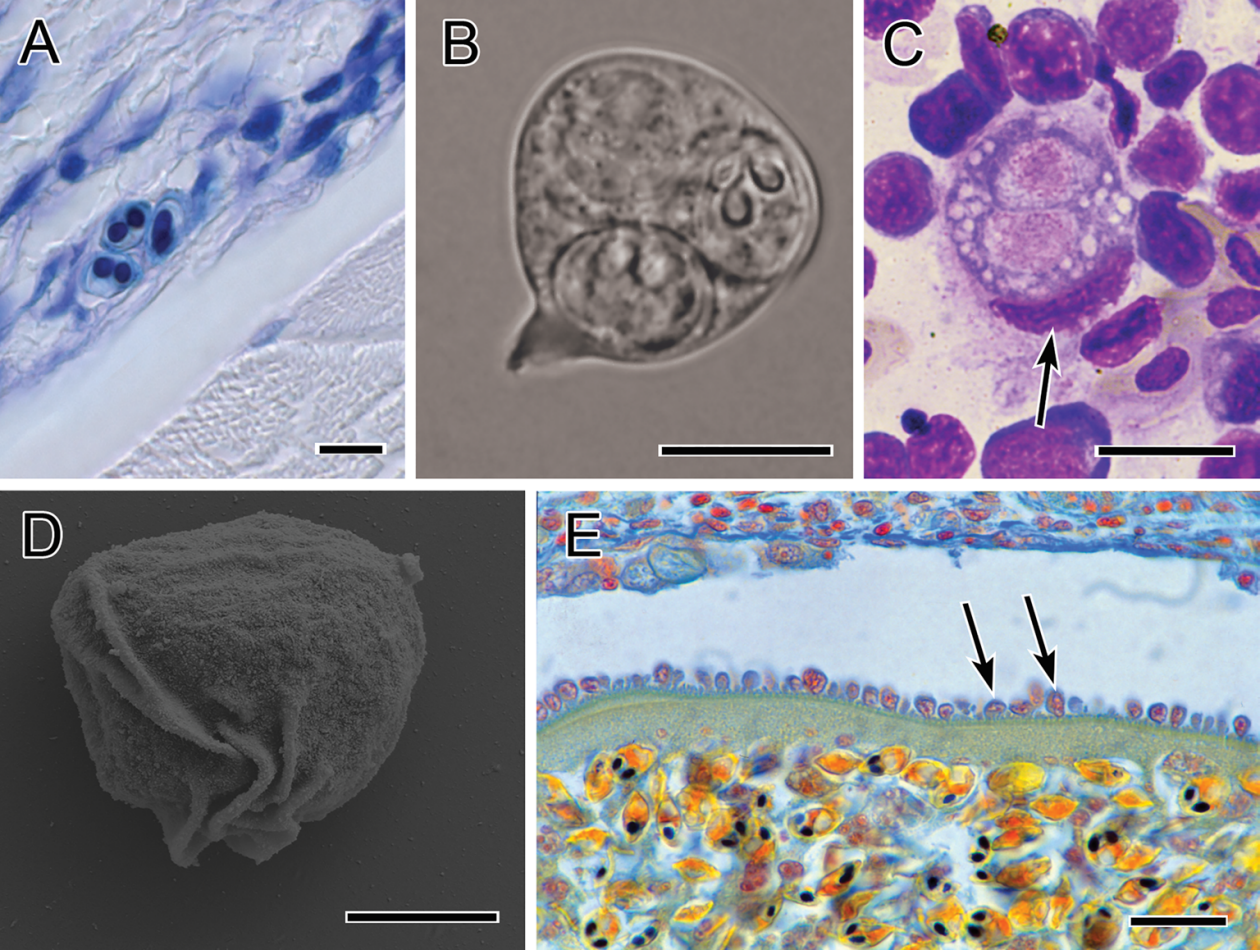
Photomicrographs showing immune evasion strategies. **(A)** Small sporogonic plasmodia of Myxobolus sp. inside a nerve strand in the muscle of brown trout (Giemsa-stained). **(B)** Plasmodium of Chloromyxum sp. floating in the bile without contact to host tissues (fresh preparation). **(C)** Typical interstitial stage of *T. bryosalmonae* in the kidney of rainbow trout, located within a single phagocyte (kidney imprint, Diff-Quick-stained; nucleus of phagocyte indicated by arrow). **(D)** Folds on the surface of a primary cell of *S. molnari* blood stage, which promote twitching motility (SEM). **(E)** Intravascular plasmodium of *Myxobolus* sp. whose outer margin is lined with host cells which are inserted into the microvillar surface of the plasmodium. Scale bars: 10 µm **(A–C)**; 5 µm **(D)**; 20 µm **(E)**. Illustration **(B)** courtesy of A Lövy, Biology Centre of the Czech Academy of Sciences. The remaining figures are from the authors.

One of the simplest immune evasion strategies of myxozoans is to simply avoid host responses by settling in organs where the immune system has a reduced surveillance capability. In this respect, the central nervous system, whose immune privilege is an experimentally defined phenomenon (162) is exploited by a number of myxozoans. Species of the freshwater *Myxobolus-Henneguya-Thelohanellus* complex (163) and the marine genus *Kudoa* infect the brain and spinal cord of fishes and form spores within nervous tissues (**Figure 4A**), e.g. *Myxobolus neurophilus* (164); *Henneguya lepturus* and *Thelohanellus lepturus* (165); *Kudoa lemniscati* (166). Infections are often without marked inflammatory response or gliosis, even when large portions of the brain (optic lobes, cerebellum, ventricle and meninges) are replaced by the parasite. Only in a few cases have pathologies such as nervous tissue compression and degeneration been reported (167). Due to their reduced immunological surveillance, nervous tissues may also provide a protected pathway for myxozoan migration to target organs. For example, *M. cerebralis* migrates along peripheral nerves to the brain and finally the skull, where spores are formed (29). Other sites within the host that are characterized by little to no host response to myxozoan infections are coelozoic habitats [**Figure 4B** (168)], represented by the bile, and renal- or seminiferous tubules. In *Sphaerospora testicularis* infections of seminiferous tubules of European sea bass, immune effectors are only activated once cysts rupture and spores spill into the interstitial tissue (169). In *T. bryosalmonae*, spore formation in the renal tubules of brown trout can continue for up to five years post exposure (160), despite acquired immunity to the parasite and its elimination from other host tissues.

Another “undercover” strategy that myxozoans can make use of because of their very small size, despite multicellularity, is intracellular development. This has been shown to occur in fast replicating, early histozoic stages of *M. cerebralis* in the epithelia and subcutaneous tissues (29, 170, 171) and likely in *C. shasta* in the endothelial cells of gill blood vessels (31). At a later stage of infection, sporogonic stages of *Kudoa* spp. [e.g. (172)] and *Myxobolus pseudodispar* (173); develop within muscle cells. It was previously believed that intracellular stages of *Kudoa thyrsites* went undetected by the host prior to myocyte rupture and release of spores (174, 175). However, this view was recently challenged by a study demonstrating early-stage detection of infected myocytes by CD8α^+/^MHIIβ^+^ dendritic cells, combined with an increased *il12* expression by activated phagocytes, even prior to spore formation (121). Another intracellular location that likely requires sophisticated immune evasion and host manipulation strategies can be found in *T. bryosalmonae.* The parasite’s characteristic interstitial cell doublets (PKX stages) are often totally engulfed by one to three macrophages. Thereby the parasite and macrophage’s plasmalemmas sometimes become interdigitated with each other, and the membranes are separated only by an intracellular space occupied by the cells’ glycocalyx (33). *T. bryosalmonae* remains intact inside macrophages (**Figure 4C**) and parasite divisions happen alongside host cell divisions (33). This likely implies that the parasite modifies the gene expression of the host cell. It has been shown that the apicomplexan *Toxoplasma gondii* survives and proliferates in macrophages only because it can manipulate host cell responses *via* GRA6-dependent NFAT4 activation, inhibiting IL12 and TNFα, suppressing nitric oxide production and downregulating MHC class II responses (176, 177). Unfortunately, nothing is known about the mechanism of manipulation of host macrophages by *T. bryosalmonae*, likely because the isolation of the host-parasite cell complexes from the interstitial tissue of the kidney has not yet been achieved.

### 5.2 Active Evasion of Host Immune Cell Contact – Motility

Motility to escape capture and recognition or clearance by host immune cells is a strategy utilized by many parasites. Myxozoans show different types of motility, from crawling and adhesion (178, 179), to nematode-like, undulating movement (180, 181) and non-directional twitching (182). Only in a few cases has the motility of myxozoans been analysed with regard to host cell interaction. In *C. shasta*, integrin-based motility promotes adhesion to the extracellular matrix of host cells, thereby allowing fast directional crawling and exploitation/destruction of host cells (179). In *Sphaerospora molnari*, the motility of proliferative blood stages facilitates feeding on erythrocytes (139) as well as evasion of host leukocytes (182). The motility of *S. molnari* arises from rapidly formed and subsequently reabsorbed mobile folds of plasmalemma (**Figure 4D**) that function like undulating membranes and results in non-directional twitching (182, 183). *In vitro* studies using immobilized vs mobile parasite stages showed that motility significantly impacts the efficacy of attachment of LPS-stimulated macrophages and granulocytes to the parasites and the rate of parasite lysis and destruction (182). The blood stream represents a common route for myxozoans to reach their target organ [e.g. (31, 184, 185)], where cellular and humoral immune effectors are abundant. *Sphaerospora* spp. proliferate in this hostile place [e.g. (18, 183, 186–188)] and since several species were reported to be mobile (187) it may be assumed that, though an energetically costly, rapid motility it is effective in avoiding contact with and attachment of host immune cells.

### 5.3 Antigen-Based Strategies

Myxozoans may purposefully mask their antigenic epitopes using host cells or proteins. Intravascular stages of *Myxobolus muelleri* in *Squalius cephalus* show host cells attached to the microvillar layer of the external plasmodial surface (**Figure 4E**), suggesting a protective role against cellular effectors of immunity in the hosts’ blood. Apoptotic cells between the inflammatory infiltrates *of E. leei* in the lamina propria have also been related to sheltering the invasive parasite stages from immune responses (189). Korytar et al. (139) observed the transport of host proteins from erythrocytes to *S. molnari* cytoplasm and cell membranes, and not just to lysosomes, i.e. cellular compartments destined to digestion, indicating a role of host proteins in parasite cells that goes beyond nutritional value. Epitope masking is the capacity of non-parasite-specific antibodies to prevent parasite-specific antibodies from binding to parasite epitopes (190). This is well known from *Plasmodium falciparum*, where IgM masking of protective IgG epitopes in infected erythrocytes is key to evading acquired protective immunity without increasing infected erythrocyte susceptibility to complement-mediated lysis (191). In *C. shasta*, monoclonal antibodies developed using ascites containing early developmental *C. shasta* stages and host leukocytes indicated that intact *C. shasta* trophozoites and trout immunoglobulin have cross-reacting carbohydrate epitopes (112), which may indicate a similar mechanism. However, it is also possible that these molecules represent parasite antigens mimicking host antigens to evade immune attacks, which has been suggested in several studies based on antibodies generated to parasite antigens in rabbits or mice, e.g. using spores of *Myxobolus rotundus* (192) and *E. leei* (193). In antigenic studies of some myxozoans the importance of glycoproteins was pointed out since they compose the predominant surface molecules and are strongly indicated to be involved in the parasite’s masking (193, 194). Furthermore, surface exposed glycans such as sialic acids may play a role in self/non-self recognition, and therefore some parasites may acquire sialic acid moieties from the host by trans-sialidases as a biological mask to evade immune detection by phagocytes or complement (193–195). However, experimental proof for host proteins used for myxozoan surface masking is lacking and the host umbrella molecules are still largely uncharacterized.

Changing antigen expression during their development appears to be one of the most important strategies used by myxozoans and has been observed in several species where parasite antigens are sequentially expressed during development, and hence are present in some stages but not in others, e.g. *K. thyrsites* (121, 196), *C. shasta* (112), *M. cerebralis* (197) and *T. bryosalmonae* (198). Antigenic variation was also suggested to occur in species where infection persists despite specific acquired immunity (118, 199). In *T. bryosalmoane* the monoclonal antibody MAb B4 was specifically bound to the secondary cell plasma membrane within extrasporogonic (PKX) stages (198). It is possible that once the fish host has generated an immune response specific to the antigens of the parasite’s primary cell, these are lysed to liberate secondary cells with a different antigenic setup. These could then survive and propagate until immunity is once again established. The Russian doll model of cell-within-cell development of myxozoans is ideal for sequential antigenic variation and, in principle, allows for 3-4 generations of cell surface changes.

### 5.4 Immunomodulation

Like other parasites, myxozoans can directly down-modulate the intensity and efficacy of host immune responses. Transcriptional studies have provided first insights into immunomodulatory pathways of myxozoans in fish. Suppressors of cytokine signaling (Socs) proteins are expressed by immune cells and cells of the central nervous system (CNS), and are important regulators of cytokine signaling pathways (200). Significant upregulation of *socs1* and *socs3* was found to be correlated with parasite burden and pathology progression in the posterior kidney of *T. bryosalmonae*-infected rainbow trout (201, 202). The expression of these molecules during *T. bryosalmonae* infection has been suggested to contribute to a general immunosuppression of the fish host. In mammals infected with *Toxoplasma gondii* and *Leishmania donovani* SOCS upregulation inhibits IFNγ-mediated macrophage activation (203, 204). In PKD, *ifnγ* expression was downregulated in the anterior kidney at 4, 6 and 7 weeks post exposure, and was accompanied with a significant decrease in myeloid cells (205). The suppression of macrophage phagocytotic activity and oxidative burst in trout infected with *T. bryosalmonae* (12) may further be related. Suppression of the *ifnγ* pathway in early infection was also reported in *Oncorhynchus mykiss* infected with *C. shasta* (114) and in *E. leei*-infected seabream (206). This indicates that *ifnγ* may be a universal target in myxozoan immunomodulation. The expression of proinflammatory cytokines in general appears to be down-regulated by myxozoans, in early-stage infections, followed by a skewed anti-inflammatory response later on, characterized by massively increased *il10* levels, e.g. in *S. molnari* (118), *C. shasta* (113, 114) and *T. bryosalmonae* (116, 154). Growing evidence from other disease models suggest that the generally protective function of IL10 can be exploited by pathogens (207) and it has been suggested that Il10 functions as a surrogate of myxozoan infections. It is possible that myxozoans induce Il10 as an immunomodulation strategy to deactivate the effector capacities of the immune system and understanding the mechanism for this may be important for controlling myxozoan diseases.

Finally, myxozoans also modulate responses of B cells. The ability to generate immunological memory can positively influence the outcome of myxozoan infections [e.g. (92, 139)]. During PKD pathogenesis, a massive activation of B cells induces hyperimmunoglobulinaemia, i.e. polyclonal expansion of diverse immunoglobulin subsets of B cells (135). Similarly, in *E. leei* infections in gilthead seabream a high Ig clonotype diversity was observed, with very few clonotypes expressed more than 50 times (93). This leads to significantly increased antibody titers, a limited proportion of which are parasite-specific antibodies. In acute *Trypanosoma cruzi* infections, hyperimmunoglobulinaemia leads not only to a dilute but also to a delayed parasite-specific antibody response and massive B cell exhaustion, consequently allowing parasite numbers to increase [reviewed by Bryan et al. (208)]. Similar effects could occur in fish infected with myxozoans inducing hyperimmunoglobulinaemia.

Immunomodulatory molecules causing shifts in cytokine expression or other immune evasion strategies can be of different classes but are surprisingly frequent amongst proteases and their inhibitors [e.g. in helminths (209),; or in trypanosomatids (210)]. In myxozoans, despite having experienced a massive genome reduction, proteases are nevertheless quite prevalent and some protease inhibitors such as serpins have greatly diversified in individual species (25). Several studies have shown that proteolytic enzymes encoded by pathogens are primary weapons of defense against host innate immunity (211). This may explain why serine and cathepsin Z-like proteases of *M. cerebralis* are up-regulated in the gills of fish during the first couple of hours post exposure to actinosporeans (212, 213). Similarly, a cathepsin Z-like protease identified in the *C. shasta* transcriptome was identified as a potential virulence factor (161). Overall, proteases and inhibitors are more highly expressed in their fish than in their invertebrate hosts (214) which may suggest key roles in immune defense, evasion, or modulation in fish (215).

## 6 Antiparasitic Strategies and Immunization

### 6.1 Effects of Diets on the Host Immune Response to Myxozoan Infection

The exponential growth of the aquaculture sector has increased the demand for fish meal and fish oil to feed omnivorous and carnivorous fish species. Clearly, using fish from fisheries to feed fish is environmentally and ecologically unsustainable, leading to the search for alternative raw materials (216). Research on plant ingredients demonstrates that replacement of fish meal and fish oil is highly feasible without detrimental effects on growth performance (217–219). However, growth performance is not the only parameter to be considered, as the nutritional background has direct effects on the immune system and resistance/susceptibility to pathogens (220). Most studies on the effect of the dietary background on the outcome of myxozoan infections have been conducted in the *E. leei* – gilthead sea bream model. Estensoro et al. (221) demonstrated that prevalence, intensity, time of acquisition of the infection and progression of *E. leei* along the intestine was worse in gilthead sea bream fed diets in which 66% of fish oil was replaced with vegetable oils. The more pronounced disease signs could not be fully explained by changes in innate parameters such as complement or production of toxic radicals (221) and one possible explanation was that replacement with vegetable oils induced changes in the goblet cell abundance and mucin composition of the intestine allowing more efficient attachment and invasion by the parasite (222). However, adaptive immunity is probably a determining factor in this interaction as Ig expression and presence of B cells were clearly affected by the diet. *IgM* transcription in the intestine was significantly higher in fish fed replacement diets, and these differences increased over the course of the infection (130, 223). These results correlated with a higher presence of IgM^+^ cells in the tissue (224). Probably, the mucosal IgT is the main player in this increased susceptibility. Upon infection, *IgT* expression in head kidney and intestine was completely suppressed in animals fed replacement diets, compared to the clear increase observed in gilthead sea bream fed control diets (130). Another study in the same host-parasite model demonstrated that the higher the replacement of fish oil with vegetable oil, the higher the susceptibility against *E. leei*, an effect that was correlated with dysbiosis of the intestinal microbiota. However, supplementation with the short chain fatty acid butyrate reversed most of these detrimental effects and ameliorated the disease signs (225). The latter results suggest a role for the intestinal microbiota in parasite susceptibility probably through microbial modulation of the host immune mechanisms, but this remains to be elucidated.

The extensive research conducted with replacement diets in the gilthead sea bream-*E. leei* model indicates that modulation of adaptive immune responses through diet is feasible, thus the use of functional feeds to control parasitic infections arises as an attractive alternative to palliate the losses in aquaculture. The use of medicated diets including anti-coccidial combinations such as amprolium, salinomycin and fumagillin were tested with different degrees of success against *E. leei* (81, 226) and *Myxobolus* sp (227). in different fish species. However, medicinal products require authorized prescriptions and detailed treatment protocols not widely available for aquaculture. Supplementation with essential oils exhibited some protection against *Myxobolus* sp., *E. leei* and *Polysporoplasma sparis* [reviewed in (228)]. Carp fed diets enriched with curcumin or a multi strain yeast fraction induced B cell proliferation prior to infection with *S. molnari* and postponed initial parasite proliferation. At later stages of infection, the anti-inflammatory effects of curcumin led to an increased parasite propagation. However, the multi-strain yeast fraction induced higher numbers of parasite-specific antibodies yielding lower parasite numbers, becoming a promising additive to decrease the impact of this disease (229). In addition, some commercially available feed additives, containing prebiotics, carvacol, thymol, organic acids, yeast or yeast extracts, demonstrated a lower intensity of infection and amelioration of the disease signs in gilthead sea bream infected with *E. leei* (230, 231). Although the anti-inflammatory and immune-regulatory effects of these diets in the fish intestine have been demonstrated (232), the mechanisms behind these effects during a parasite infection are still unknown. To date, complete protection against myxozoan infections by diet interventions has not been achieved and, although some effects can be observed, it remains difficult to translate nutritional prophylactic strategies into immediate solutions for complex, multifactorial processes across different host-parasite models.

### 6.2 Acquired Immunity Against Myxozoan Infections: Paving the Road Towards the Development of Anti-Myxozoan Vaccines

The development of a functional vaccine depends on the activation of a specific immune response that will eventually confer protection against infection. Protective acquired immune responses against several myxozoans have been demonstrated in different fish species. In natural infection scenarios, Atlantic salmon and Atlantic cod showed acquired resistance to *Parvicapsula pseudobranchicola* and *Gadimyxa atlantica*, respectively, upon secondary or continuous exposure to the parasites (233, 234). However, the immune mechanisms behind this protection were not investigated. Other studies using experimental infections, revealed that the acquired protection against myxozoans is, at least in part, related to the production of specific antibodies. Rainbow trout that survived infection with *T. bryosalmonae* were re-infected one year later, and, even though they were still susceptible to infection, lower prevalence of infection and limited pathology were observed and correlated with higher numbers of IgM^+^ B cells in blood and soluble IgM transcription in kidney (205). Protective immunity in Atlantic salmon against *K. thyrsites* was shown to be mediated by activation of antigen presenting cells (macrophages/dendritic cells), cytotoxic T cells and IgM and IgT expressing B cells (121). Turbot re-exposed to *E. scophthalmi* presented no disease signs or death even after 3-4 years of the first exposure. This resistance was correlated to a higher presence of parasite-specific IgM in serum, particularly between 50 and 150 days after exposure, but the levels of mucosal IgT were not evaluated for this intestinal parasite (140, 141). The protective immune response elicited by a similar parasite, *E. leei*, in gilthead sea bream has been studied in more detail. *Enteromyxum leei*-specific antibodies (IgM) were detected in high levels in serum of gilthead sea bream that had survived and cleared a previous infection, and directly correlated with complete protection against re-infection. This protection lasted at least 16 months, and re-infected animals showed higher expression of *IgT* and *IgM* together with a post-inflammatory transcriptional profile in the intestine (92). Levels of parasite-specific IgT were not determined, but immunoglobulin repertoire analysis revealed that certain IgT clonotypes were being overexpressed at the infection site in re-exposed gilthead sea bream, highlighting the importance of this mucosal immunoglobulin in the interaction with this enteric parasite (93). It is important to point out that, due to the difficulty to standardize infection procedures, some of these results have been extracted from natural infections where animals are under continuous parasite pressure at certain times of the year, whereas others come from a single controlled exposure in the lab. Laboratory exposures also vary, and in some models a single exposure to the pathogen is enough, whereas in others the exposure can last several days or even months. The effect of all these variables on host protective responses remains to be studied.

The development of vaccines against myxozoans is still nascent. However, research towards immunization against these parasites has been conducted. For instance, a low magnitude specific humoral response was achieved when European sea bass were intracoelomically injected with sonicated *Sphaerospora dicentrarchi* myxospores in combination with Freund’s complete adjuvant. The number of parasite-specific-secreting cells was increased in the head kidney, but serum specific antibodies could not be detected (235). In any case, myxospores are produced at later phases of the infection, thus their use as vaccine candidates is probably not the best choice, and while infective actinospores or proliferative stages are better candidates to search for vaccine targets, they are difficult to isolate in large quantities. However, the establishment of the life cycle of *M. cerebralis in vivo* has enabled testing the effectiveness of UV treatment on waterborne infective stages, or triactinomyxons, as an attenuated vaccine in juvenile rainbow trout. Different doses of radiation were tested and positively correlated with higher inhibition of the parasite’s life cycle completion, none of them preventing the key steps in the initiation of parasite infection. Thus, lower radiation doses resulted in activating the adaptive immune responses of the host without long-term survival of the parasite, inducing protection to re-infection, at least, up to 5 months post first exposure (236). An approach similar to an attenuated vaccine was performed with *C. shasta* in Chinook salmon, where animals were exposed to a non-host specific genotype of the parasite (genotype II) for 24 hours, and 53 days later challenged with the host-specific genotype I. Unfortunately, no protection occurred in this trial (237). Further testing with different timing or doses of exposure might yield different results, but possibly this lack of protection is due to antigenic differences among genotypes.

The design of more sophisticated vaccines against myxozoans, such as subunit or DNA vaccines, has been delayed mainly due to the lack of molecular information on the parasites. Generally, myxozoans are difficult to separate from host tissues and difficult to purify in large quantities, hindering the construction of good quality genomes and transcriptomes. However, the design of optimized pipelines to deal with host-parasite mixed samples (134, 161), together with the advent of high-throughput sequencing technologies, is allowing for the generation of genomic and transcriptomic information of myxozoans. To date, transcriptomic and/or genomic assemblies are publicly available for a limited, yet continuously growing, number of species [Reviewed in (25, 215)], opening up possibilities for exploitation of this information to produce anti-parasitic tools. For instance, the use of virulence factors, such as serpins, to develop anti-parasitic therapies has been recently proposed (25). All these new resources are currently being used in the search for vaccine candidates based on *in silico* searches for virulence factors, extracellular proteins or yet uncharacterized proteins that might have antigenic potential. Due to the complex life cycle of these parasites, an important feature of a good vaccine candidate is its high expression in the parasite stages present in the fish host. Genomes may contain some information only expressed in the invertebrate host which might not serve to induce protective immunity against the fish proliferative stages. Following this rationale, a recent work compared the transcriptomic profiles of *T. bryosalmonae* in its fish and bryozoan hosts (214) allowing for the shortlisting of vaccine candidates to be used in rainbow trout. As a follow-up of that work, several identified vaccine candidates have been tested in DNA vaccination trials, inducing partial protection and reducing PKD-associated pathology and parasite load. Among the selected candidates, the most promising antigen was a novel micro-exon gene expressed in and on the surface of the parasite, which demonstrated a potent specific antibody (IgM) response in rainbow trout sera after vaccination (238). It is expected that within the next few years, novel candidates for other myxozoan species will be tested in order to develop effective vaccines to be used in aquaculture.

## 7 Final Remarks and Future Perspectives

The evolutionary origins of Ig-producing B cells appear to be linked to the emergence of fish on Earth while myxozoans evolved long before these basal vertebrates, as parasites of invertebrates (239). Upon conquest of the new host, myxozoans had to adapt to surviving in an environment equipped with advanced pathogen-specific defense mechanisms that include immunological memory. Myxozoan-specific antibodies were first demonstrated in the 1990s and our knowledge of fish immune responses to myxozoans has advanced considerably since, primarily due to gene expression studies. However, very little is known e.g. about immunological memory and the mechanism thereof.

To date, few functional studies on immune reactions to myxozoans have been conducted due to a lack of tools, especially fish leukocyte-specific antibodies. Antibodies are generally produced in warm-blooded mammals and it is likely that some of the problems related to their design are based on differential protein structure and folding at higher temperatures. The development of synthetic aptamers hence represents promising perspectives.

On the other hand, the fast rate of evolution of myxozoans and the lack of isolation and *in vitro* culture models considerably complicates characterizing host-parasite interactions, parasite antigens or adaptive and immunomodulatory mechanisms. There are very few experimental parasite models in place, and maintaining these is both labor- and time intensive, as the life cycle in both hosts usually takes about 6 weeks to be completed. Furthermore, migration routes of early parasite stages remain to be resolved, as missing links exist for most species between the portals of entry and the target tissues. Future efforts should focus on methods for isolating and culturing/cryopreserving infective spores, their sporoplasms or early proliferative stages to use in season-independent *in vivo* and *in vitro* trials. Host-free isolates of myxozoan pre-spore stages are required to study the interaction with host cells, ideally using single cell sequencing approaches, which allow analyses of cellular responses. Importantly, host and parasite strategies should be analysed simultaneously to understand how specific immune responses are modulated by changes in the transcriptomic response of the parasite and vice versa. Changes induced in macrophage and lymphocyte gene expression upon exposure to parasites could e.g. shine a light on the modulation of Ifnγ and Il10 pathways as immune evasive strategies and help identify modulator molecules used by the parasites.

In the light of the present progress of climate change, there is a requirement for rapid development of effective anti-myxozoan therapies, and strategies based on immune stimulation will unlikely be sufficient. The importance of antibodies to the course of myxozoan infections has been demonstrated in several species. It is hence timely to take the next step and characterize immunoprotective antigens as a basis for future vaccine design. Promising perspectives exist regarding the application of such antigens as DNA/RNA vaccines, which have been the focus of aquaculture vaccine developments over the last 20 years (240). Genomic and transcriptomic datasets of a larger number of myxozoan pathogens are required to compare immune responses between and among species and to identify trans-species vaccine targets for the aquaculture sector. Regarding the host perspective, exploitation of resistant species and strains has applications in disease management and would benefit from further immunological characterization of these resistant hosts.

## Author Contributions

All authors contributed to the article and approved the submitted version. Conceptualization, writing of original draft, reviewing, and editing – all authors. Visualizations – AS-B and AH. Extended language editing – JB. Formatting – MCP.

## Funding

MCP was funded by a Ramón y Cajal Postdoctoral Research Fellowship (RYC2018-024049-I/AEI/10.13039/501100011033 co-funded by the European Social Fund (ESF) & ACOND/2020 Generalitat Valenciana). JLB and DB were funded by the Bureau of Reclamation, U.S. Department of Interior through Interagency Agreement #R19PG00027. The funders had no role in study. AH and the open access publication of the present article was funded by the Czech Science Foundation EXPRO grant #19-28399X (AQUAPARA-OMICS; 2019-2023). Part of the information gathered in this review was obtained with financial support from the European Commission under the project #634429 (ParaFishControl).

## Author Disclaimer

This publication reflects only the authors’ view, and the European Union cannot be held responsible for any use that may be made of the information contained herein.

## Conflict of Interest

The authors declare that the research was conducted in the absence of any commercial or financial relationships that could be construed as a potential conflict of interest.

## Publisher’s Note

All claims expressed in this article are solely those of the authors and do not necessarily represent those of their affiliated organizations, or those of the publisher, the editors and the reviewers. Any product that may be evaluated in this article, or claim that may be made by its manufacturer, is not guaranteed or endorsed by the publisher.
